# Attitudes, understanding, and concerns regarding medical research amongst Egyptians: A qualitative pilot study

**DOI:** 10.1186/1472-6939-8-9

**Published:** 2007-08-29

**Authors:** Susan S Khalil, Henry J Silverman, May Raafat, Samer El-Kamary, Maged El-Setouhy

**Affiliations:** 1 Ain Shams University, Cairo, Egypt; 2 University of Maryland School of Medicine, Baltimore, Maryland, USA

## Abstract

**Background:**

Medical research must involve the participation of human subjects. Knowledge of patients' perspectives and concerns with their involvement in research would enhance recruitment efforts, improve the informed consent process, and enhance the overall trust between patients and investigators. Several studies have examined the views of patients from Western countries. There is limited empirical research involving the perspectives of individuals from developing countries. The purpose of this study is to examine the attitudes of Egyptian individuals toward medical research. Such information would help clarify the type and extent of concerns regarding research participation of individuals from cultural, economic, and political backgrounds that differ from those in developed countries.

**Methods:**

We conducted semi-structured interviews with 15 Egyptian individuals recruited from the outpatient settings (public and private) at Ain Shams University in Cairo, Egypt. Interviews were taped, transcribed, and translated. Thematic analysis followed.

**Results:**

All individuals valued the importance of medical research; however most would not participate in research that involved more than minimal risk. Individuals were comfortable with studies involving surveys and blood sampling, but many viewed drug trials as being too risky. All participants valued the concept of informed consent, as they thought that their permission to be in a research study was paramount. Many participants had discomfort with or difficulty in the understanding several research concepts: randomization, double-blind, and clinical equipoise. Trust in the physicians performing research was important in deciding to participate in clinical research. The small sample size and the selection bias associated with obtaining information from only those who agreed to participate in a research study represent limitations in this study.

**Conclusion:**

Overall, individuals in our sample recognize the value of medical research and have a great deal of trust regarding medical research and their participation in research. There were, however, concerns with the level of research risks associated with several types of medical research. Many also demonstrated confusion with certain research methodologies. We recommend 1) enhanced educational efforts regarding general research concepts to enhance the validity of informed consent and 2) further survey studies in other areas of Egypt to determine the generalizability of our results.

## Background

Medical research involving human participants has increased greatly in many developing countries during the recent decade, motivated by the need to address the high burden of diseases in these countries. The ethical conduct of research specific to developing countries has been the subject of recent discussions [1-3] and has been addressed in several research ethics guidelines [4,5]. Issues raised by these publications have included the relevance of the research to the health needs of the community and the country, avoidance of exploitation, and assurance that the informed consent process is sensitive to the local context, yet also representative of genuine, independent choice.

Since medical research requires the participation of human subjects, high levels of patient recruitment are required for a successful research agenda. Factors that might affect willingness to participate in medical research include patients' perceived benefits associated with their participation in drug trials [6,7], confusion with research methods such as blinding and randomization [7,8], the level of patient understanding of information about the research [9], and the level of trust patients place in investigators [9]. Knowledge regarding the understandings, perspectives, and concerns of individuals with their potential involvement in medical research would be important with improving recruitment efforts, enriching the informed consent process, and enhancing the overall trust between investigators and the public. Such knowledge would also help with devising strategies to improve communications between patients and investigators.

Several qualitative studies have been performed eliciting the views of patients regarding medical research from the United States, Denmark, Australia, and Japan [10-13]. Such results, however, might not be generalizable to developing countries that incorporate different ethnicity, religions, cultures, economic, and political backgrounds. Currently, there is limited empirical research involving the perspectives of individuals from developing countries [14,15] and from countries in the Middle East. Additional studies would be helpful in further clarifying concerns, misperceptions, and underlying themes regarding research participation of individuals from these countries. Accordingly, we conducted a pilot, qualitative study involving individuals living in Egyptian society regarding their perspectives on medical research. We expect that information obtained from this study would be helpful to individuals involved with research in developing countries, in general, as well as those involved with research in Egypt and other Middle Eastern countries. A long-range goal is that our results will be instrumental in designing further comprehensive quantitative survey studies in the international context.

## Methods

### Setting

We recruited a convenient sample of 15 individuals from the outpatient clinic waiting areas from two of Ain Shams University hospitals: Ain Shams University Specialty Hospital, a semi-private university-based hospital, and Ain Shams University Public Hospital, a public teaching hospital. Both hospitals are situated in the metropolitan area of Cairo and serve as referral hospitals for patients predominantly coming from northern (Lower) Egypt: Cairo and areas of the Nile Delta, which fans out north of Cairo to the Mediterranean coastline to include Alexandria and Port Said. Urban life in Lower Egypt is marked by some 20–30 percent of the population living below the poverty line and an illiteracy rate of approximately 55 percent. Amidst the poverty, uneven development has led to extremes in the distribution of wealth, as evidenced by the emergence of an affluent class that stands in contrast to a significant number of poor urban Egyptians who live in overcrowded housing and inadequate access to clean water, good quality health care, or education. Historically, Lower Egypt has been more prosperous than the predominantly rural Upper Egypt, which extends from 120 kilometers south of Cairo to the border with Sudan. One third of the population and half of Egypt's poor live in Upper Egypt and it is also the area with the highest infant mortality rates, 36 percent above the national average. Ethnic minorities in Egypt include the Bedouin Arab tribes of the Sinai Peninsula and the eastern desert, the Berber-speaking community of the Siwa Oasis and the Nubian people clustered along the Nile in the southernmost part of Egypt [16].

### Recruitment

Potential participants for our study were recruited from the clinics' waiting rooms; they were either waiting for their clinic appointments or were in the clinic area accompanying their family members who had clinic appointments. Two of the authors (SSK and MR) approached individuals and briefly informed them of an interview study involving determining individuals' attitudes towards and experiences with medical research. If individuals expressed interest, they were accompanied to a private room near the clinic waiting areas where they received further information about the study. If they agreed to be interviewed, then verbal consent was obtained and documented on the audiotape. The small sample size and the selection bias associated with obtaining information from only those who agreed to participate in a research study represent limitations in this study.

### Interviews

Two of the authors (SSK and MR) received training in interviewing techniques and used a semi-structured interview guide that elicited open-ended responses from 15 research participants. All interviews were conducted in colloquial Arabic. The interviewers were medical students at Ain Shams University, who had no prior relationships with the individuals recruited for the study. Interviews lasted approximately 45 minutes and were audiotaped. All of the interviews were conducted during a period of six months. Audio recordings were transcribed in Arabic and then translated into English. Interviewers helped participants complete a form that collected demographic information.

### Semi-structured interview tool

The survey tool consisted of open-ended questions that assessed the attitudes of the individuals regarding the following broad categories:

• medical research and their willingness to participate in different types of research

• studies with different types of risks

• research concepts (e.g., randomization, double-blinded, equipoise)

• informed consent

• motivations of researchers

We were aware that the different words used to describe research might convey very different meanings and hence, might play a role in how individuals understand and perceive the concept of research. Indeed, a previous study showed that the term "medical study" was viewed as being more positive and benign than the term "medical experiment."[10] When interviewers asked participants how they felt about research, interviewers initially used an Arabic phrase ( abhas tibbiya) that denoted *medical study or research*. However, it became apparent that many participants equated this phrase with benign studies, such as survey research. Consequently, interviewers used another Arabic phrase ( tagriba tibbiya) that conveyed the meaning of *medical experiment*, which helped ensure that participants understood that other types of studies, such as drug trials, were the focus of the interview. When appropriate, interviewers explicitly explained the types of research studies that were the subject of the questioning.

### Confidentiality

Participants were assigned a unique name different from their actual name. These names were used during the interview. This unique name was coded to the individual's demographic form, which did not use the individual's real name. After the tapes were transcribed they were destroyed.

### Analysis

An emergent coding approach was used, whereby two of the authors (SSK and HJS) independently analyzed the content of the transcribed texts to identify patterns and themes that emerged from the data [17]. Themes and sub-themes were segmented into categories according to a consensus-driven coding scheme. In the final stage of analysis, a matrix was developed to compare major themes and patterns within and across interviews [18].

### Research Ethics Committee approval

The research ethics committees at Ain Shams University and the University of Maryland, School of Medicine gave approval for the conduct of this study and approved the mechanism of obtaining verbal consent.

## Results

### Demographic and background information

Of the 15 participants, 10 were female and 5 were male. Nine were recruited from the private hospital and six were recruited from the public hospital. Thirteen of the participants were from Lower Egypt, whereas the two resided in Upper Egypt. The age range was from 19 to 69, with a median age of 30 and an average age of 38 years. Three participants had completed high school, eight had an undergraduate college degree, and four participants had attained a degree higher than undergraduate level. Of these participants, eight were patients waiting for their clinic appointments, while seven were accompanying family members to the clinic. Eight participants were married, all with children, while seven participants were single. All but one participant was unemployed; nine were considered to be in the lower income bracket (less than 1000 Egyptian pounds/month or less than $175/month total household income). For each participant who agreed to participate in the interview study, approximately four were approached. Common reasons given for refusal to participate in the study included time constraints or fear of missing their appointments.

### Description of findings

We identified several major themes from the analysis of the data; these are listed in Table 1.

**Table 1:** Themes and sub-themes identified by analysis of interviews

**Perception of medical research**
Good understanding of clinical research
Positive attitude towards motivations of doctors performing research
Positive attitude towards research participation
**Importance of risk in the decision to participate in research**
Important of the degree of risk in determining willingness to participate in research
Willingness to participate in research decreases as the risk level of the study increases

**Beliefs regarding benefits derived from research participation**

**Understanding of research concepts in a clinical trial**
Discomfort with randomization
Variable understanding of concept of equipoise
Discomfort with doctor not knowing which drug is better
Discomfort with doctor being blind to treatment assignment

**Issues with informed consent**
Informed consent is an important value
Little discomfort with written informed consent
Mixed acceptance of proxy consent
Mixed reactions toward giving permission for children participation in research

**Trust is an important value for patients**

**Concern with the prospect that research involves experimentation**

### Perception of medical research

#### Good understanding of medical research

All participants had a good understanding of medical research as a means of finding out more information about a certain topic. For example, one male patient from the private clinic said *"In order for a theory to be proven there must be evidence to substantiate it." *A woman from the public clinic said "*In a research study we focus on a specific problem and begin to or try to research it from different angles until we reach a certain result."*

In response to being asked "Why is research important?" several individuals mentioned that medical research is important to "advance the field of medicine," it can "find cures," "create medications to treat [diseases]"' and it "generates new discoveries." One participant said *"...this is an age of emerging diseases and they have to find cures for them," *while another participant said "*It's a means to study the patient ...so that they can make the proper drug*."

#### Positive attitude towards motivations of doctors performing research

When we asked participants what reasons motivated doctors to do research, all said that they thought doctors wanted to advance medicine, to find new treatments, obtain new information, or to improve patient care. When we asked if they thought that doctors do studies only to promote their careers, nine of the participants accepted the possibility that doctors do research to advance their careers, but eight of these nine participants did not believe career advancement was the sole reason for doing research and that doctors are also doing research to advance medicine and to help other patients.

#### Positive attitude towards research participation

Most participants (13 of 15) reported they would agree to participate in a study even if there weren't direct benefits to them. Eight of the respondents stated that their reasons for participation would be the chance to help other patients. One participant stated: *"Yes it [medical research] is important, because it's beneficial. If it won't benefit me, it can be of benefit to others." *Another one stated: *"It doesn't have to necessarily benefit me, but if it may help someone else I would agree to participate." *Another respondent stated: "*Yes, of course [I would agree to participate even if there weren't any direct benefit] because the individual does not live alone, but is rather a link in a large chain."*

### Importance of risk in the decision to participate in research

#### Importance of the degree of risk in determining willingness to participate in research

While the participants were willing to participate in research even if there were no direct benefits, five of these participants qualified such willingness based on the existence of side effects, specifically mentioning their reluctance to participate in drug studies. For example, a middle-aged woman attending the public clinic said; "*But a clinical trial would be something that's a little too difficult*."

#### Indeed, when we asked the participants

"How important are the risks to the study in deciding whether or not to participate in a study?" all except one respondent reported that the risks of a medical study would influence their decision to participate. While three of the respondents would not participate if there were any risks involved, eleven qualified their answers. For example, five respondents mentioned their fear of being in drug studies or studies that will affect their health or lead to physical harms. Two respondents distinguished between the types of risks involved. For example, one respondent said "*It's not such a problem if it's an economic risk or an emotional risk, but a health risk, that's difficult*." Another respondent told the interviewer "*I'll take the questionnaire, but I don't want to be ...experimented on....it's guinea pigs that you experiment on*." One participant mentioned the importance of weighing the risks and benefits and making sure that the benefits are greater than the risks, another stated that "*there are limits to any risks*" and another respondent stated "*It must have some guidelines that guarantee that it will protect me."*

#### Willingness to participate in research decreases as the risk level of the study increases

To obtain a better understanding of the role of risk in willingness to participate in medical research, we gave the participants examples of medical studies that involved procedures with varying degrees of risk. We used studies that ranged from a minimal risk level to higher risks. These included a blood sampling study, a survey study that inquired about sensitive information (such as medical condition, income level, etc), a study requiring a biopsy of the skin, a study requiring endoscopy, and a study involving the administration of an experimental drug. The aggregate results are shown in Table 2.

**Table 2:** Relationship between the type of study and willingness to participate in the study. Studies are listed in order of increasing risk levels

**Type of study**	**Willingness to participate**
Blood sampling study	13/15
Survey study involving sensitive information	12/15
Skin biopsy study	3/14
Endoscopy study	1/14
Drug trial	5/15

#### a. Blood sample study

Most participants (13/15) agreed they would give a blood sample in a study that would not directly benefit them. Two respondents emphasized that giving blood is a normal activity in the clinic. Of the two participants that objected to a blood sampling study; one stated "*I'm afraid of diseases" *and the other said "*I'm worried if a study requires experimenting on me in particular...It's like I said, you usually won't resort to entering a study or a clinical trial unless you feel like there's something that you may gain from being in it...I can't see it helping me."*

#### b. Survey study requiring sensitive information

Almost all participants (12/15) reported they would agree to participate in a survey study that asked for sensitive information, even if there weren't any direct benefits involved. One respondent stated: *"If there are personal experiences, I won't be shy to share this. I mean even if it is personal, but it may be of benefit to the study–or they'll find some treatment for this or if they need to know it to find its underlying reasons or something...then I'll say it*. Another told the interviewer: "*Because if everyone of us just hides what they find to be private, there won't be any studies. There won't be anything new, we won't be able to find out what's wrong with us and remedy [fix] it. So I would share." *One respondent from the private clinic qualified her answer by saying "*Most studies don't tie the information collected to any identifying information. So I don't think I would mind this*." Three reported that they would not agree. One participant explained: *"Sometimes there are things that are too personal that are hard to share." *Another stated:*"No, I wouldn't. Since it won't be of any benefit to me. And I don't know, I wouldn't feel comfortable answering their questions."*

#### c. A study requiring a biopsy of the skin

Participants' reactions were mixed regarding their participation in a study involving a skin biopsy. Eight reported that they would agree to such a study, although five of these participants qualified their willingness to participate by stating if the study did not involve too much risk. For example, one stated: *"I don't see any major risks in something like that...I wouldn't participate if there were risks," *while another said *"It depends on where it's from. If it doesn't involve any risks then I'll agree." *Six participants reported that they would not agree to a biopsy for fear of the risks involved. Responses included: *"That sounds more dangerous of course" *and *"No, that's a little too difficult to endure, it worries me. Anything that involves some kind of surgical procedure worries me, even if it's a good physician." *One participant did not give an answer to this question.

#### d. A study requiring an endoscopy

The majority of the participants reported that they would not agree to a study requiring an endoscopy. The main reason given for refusal was due to the risks involved. One individual stated: *"No, that sounds like too much to endure. God forbid, that's a procedure that's very invasive." *Another explained: *"No." "Because I hear that endoscopy has many risks involved." *Finally, one individual stated: *"No [I wouldn't agree to participate in such a study]...I hear that sometimes the instrument isn't good, and it may be contaminated so it may cause some problems." *The two respondents who would agree to participate in such a study qualified their willingness by saying "*only if it doesn't have any risks*" or "*if the doctor is someone that I've known for a long time....then it would be okay*."

#### e. Clinical trial involving an experimental drug

We asked participants their willingness to participate in a drug trial that compared an experimental agent with an approved drug on the market. We also said that that the balance of risks and benefits of the experimental agent is believed to be similar to that of the approved drug – concept of clinical equipoise. While we were able to distinguish between respondents willing or not willing to participate in such a study, many of the responses we received indicated confusion with the basic underlying concept of a drug trial. For example, while six of the 15 participants expressed their willingness to participate in such a study, two of these participants said they would participate only if experimental agent is thought to be better than the approved drug. One said "*if my doctor says it's better, then I'll try it*." While the other respondent said "*but the new drug must have a better impact than the old drug*." Two participants agreed to participate in the trial anticipating better results from the newer drug, although they did not need a guarantee of better results, as the other two participants. Of the eight who would refuse participation, three could not understand why anyone would enroll in such a trial if there was already an approved alternative on the market. Two respondents expressed being scared of drug studies, one did not want to be "experimented on," one stated he had allergies, and another respondent doubted the concept of equipoise: "*How do you know that it has the same risks, when you still haven't tried it on people yet? I can't go on someone's assumptions and expose myself to such risks*." One last respondent's answer indicated he did not understand the concept of a drug trial.

### Beliefs regarding benefits derived from research participation

We did not ask the participants specifically what they thought about the prospects of obtaining benefits in medical research. However, in response to several of the questions (e.g., regarding being in a drug clinical trial, proxy consent when critically ill, and enrollment of children), several individuals voiced a belief that medical research could lead to benefits. For example, in response to being asked to participate in a drug clinical trial, one respondent said: "I definitely would agree to participate, but the new drug must have a better impact than the old drug." In response to allowing proxy consent when critically ill, an individual said "I'm sure that they'll do what's in my best interest, so yes." Finally, in response to being asked regarding enrollment of children, one person said: "There has to be mutual benefit," while another respondent said "If I feel that the study will be of benefit."

### Understanding of research concepts in a drug trial

#### Discomfort with randomization

We inquired as to how participants felt towards the process of randomization. We asked them how they would feel if treatment assignment in a drug trial would be determined by a coin toss, hence giving them a 50/50 chance of receiving either the experimental agent or the approved drug. Twelve participants felt uncomfortable with accepting such a process and many of these did not seem to understand the concept. Several participants reported feeling worried and that this process might involve more risk. One respondent said *"No, I'm not comfortable with it. He [the physician] should tell me what the risks and benefits of each drug are and I should have the freedom to decide which of the drugs I want [I'll take]." *Another stated *"I don't like the idea of it being random. I know that one that's on the market is available in the pharmacy, and there's the newer one. I'll just take the older one." *Another one said: *"This random selection method is not guaranteed [safe] at all...It's like I said, it's just not safe. I just can't see how it can be safe to randomly assign something in medicine [medical care]." *Three participants did not have difficulty with such a selection process. These participants had varied backgrounds in terms of age, educations, and type of clinic they were attending (public vs. private).

#### Variable understanding of concept of equipoise

After telling the participants again that there is uncertainty of the relative risks and benefits between the experimental agent and the approved drug and that the aim of the clinical trial was to determine which drug is better, we asked them if it mattered which intervention they received. Eight participants had no preference, three preferred the new drug, two preferred the existing drug, and one participant stated a preference for the drug that had the least side effects.

#### Discomfort with doctor not knowing which drug is better

We asked participants their thoughts regarding that physician-investigators are uncertain of which drug would be best for them. Most participants (12/15) reported discomfort with such uncertainty. Many expressed disbelief that the physician-investigator would not know which drug is better for them. For example, one respondent said: *"I don't know, I'd be very surprised how my doctor doesn't know, I might be worried how he's a doctor and doesn't know. He's specialized in this and doesn't know, so how should I feel but worried." *Another one said: *"Uncomfortable is not a term that's enough to describe how I feel...I go to the doctor with my health in their hands, which is the most precious thing to me. The fact that there's some knowledge that they lack, which will affect my health, I see that as a big problem." *Another one said: *"I'll be very irritated because the doctor must be aware of everything," *and another said "*If the doctor doesn't know, of course I'm going to be worried*."

#### Discomfort with doctor being blind to treatment assignment

We asked the participants how they would feel about the physician-investigator not knowing to which drug that they would be assigned. Five respondents would not accept to be in such a study: three said they needed to know which drug they were receiving, while the other two stated that their doctor had to know which drug they were receiving. Responses from five respondents indicated they lacked an understanding of the physician-investigator being blinded to the treatment option. Three participants said they would participate in such a study, but would be either "worried" or uncomfortable with such a situation. Two respondents would agree to participate in such a study, concluding that this study made them feel more comfortable about the results that would be generated, since the results wouldn't be biased. For example, one stated *"I'm more comfortable in a situation where the conclusion was reached in a logical way. Where there isn't external influence, not from the physician, not from the company, but from my personal experience [with the drug]" *while the other respondent said: *"I think I might have more trust in his opinion as a result, since he won't be influence"*

### Issues with informed consent

#### Informed consent is an important value

We asked participants if it were important for them to give their permission to be in a research study. All participants valued the importance of informed consent and some framed their answer in the language of rights. For example, one respondent said: *"Definitely, it's important to respect the rights of a patient." *Another individual said: *"Yes, definitely, since you have to ask the patient first and everyone has this right." *One individual told the interview: *"Yes, of course...Aren't I the one that's going to join with my body and my health? I mean, who else would they ask."*

We also presented to the participants a scenario of an investigator wanting to do a research study with the use of the left-over blood from their blood samples that had been obtained for clinical purposes. We asked if it would be appropriate for the investigator to use the blood without their permission. Of the15 participants, 13 said it would be okay mainly because the blood had already been taken.

#### Little discomfort with written informed consent

We told the participants that for the current interview study, they only had to give verbal consent, but for many other types of studies, an investigator would probably ask them to sign documents stating they had given informed consent. We asked them how they would feel with signing such documents. Of the 15 participants, 13 felt comfortable with giving written informed consent. However, two respondents had hesitation with signing their names, both were women in their thirties and both were at the public clinics. One of these participants, who had a bachelor's degree, was afraid that their signature would mean that he or she would feel obligated to continue in the study despite a desire to withdraw. The other individual, whose highest level of education was high school, was afraid that the document would "fall in the wrong hands."

#### Mixed acceptance of proxy consent

We asked participants if they would agree to have a family member give proxy consent for their participation in a study if they were critically ill and could not make decisions for themselves. Ten participants reported they would not agree to proxy consent. Six were women of whom five were from the private clinics and four were married. Of the four men who were refusing proxy consent, one was from the private clinic and three were from the public clinic. A variety of reasons were given for such refusal. For example, one participant expressed concern about being enrolled in a study when critically ill:*"No, it has to be me [who gives consent]; not even my husband." *Another participant said: *"I'd like it to be my decision, so that I take full responsibility for whatever the outcome is." *Finally, another person said "*Too ill to speak? I'm in a coma? No, I won't agree*."

In contrast, five participants would agree with proxy consent; four were women and one was a man. One participant said "*I'm sure that they'll do what's in my best interest, so yes*," while another said "*Yes, it's okay for them to ask my husband or my children*."

#### Mixed reactions toward giving permission for children participation in research

We asked the participants if they would allow their children to participate in research. Six of the 15 respondents were not agreeable to such a concept. Four of these respondents were women (three single, one married), while two were men. Of the nine participants who said they would agreed to enroll their children in research, five (all women), specifically mentioned that their permission would be conditional. For example, three stated the study would need to be associated with benefits for their children. Another said that she would "be hesitant if it has risks" and another respondent mentioned that there needs to be assurances that the study would not involve experimenting on her child. Hence, of the 11 respondents who either would refuse enrolling children research or would conditionally enroll, nine were women.

### Trust as an important value

Throughout the interviews, the theme of trust emerged as an important value for individuals when considering participation in research. For example, several respondents mentioned that for them to participate in research, they would need to trust the physician asking them to enroll in the research. One respondent stated: "*I have to be able to feel like I can trust them. As soon as I'm certain that I can trust them, then I'd be able to agree to participate*." Another respondent said: "*I think I'd be more likely to agree if my physician asked me...If I can't trust my physician then who can I trust?" *Another participant, a nineteen-year old female college student, reported the importance of physicians' involvement in the research, since she would trust them more than other health care professionals.

In response to a study involving endoscopy, one participant stated: *"No, I'd be afraid...If it's a doctor that I've known for a long time... then I might agree to the endoscopy... [If it was a doctor that I did not know well] It would depend on how much I trust them"*

When asked about participating in a study in which the risks and benefits of the experimental drug was similar to that of the approved drug, one respondent said "*I'll participate if my doctor says it's okay for me to participate.... I trust my doctor one-hundred percent*."

When asked about participating in a study in which physicians would be blinded to the treatment assignment, a respondent mentioned that whether or not he would be willing to enroll in the study would "*depend on how much I trust them*." Another respondent stated: *"I'll trust the physician if he explains this to me first, it will make me feel more comfortable*."

### Concern with the prospect that research involves experimentation

Throughout the interview, participants mentioned many times their thoughts that research is an enterprise that can be associated with experimentation, a concept that instilled fear into them. The mention of such statements transcended age, gender, and clinic type. For example, several respondents viewed drug trials as akin to experimentation. A 26 year-old male from the public clinic mentioned "*I'll take the questionnaire study, but I don't want to be in a situation where I'll be experimented on. First of all, it's guinea pigs that you experiment on*." A 69 year-old male from the private clinic mentioned that "*I may participate in [a study] with an exception of those that involved experimenting with drugs on me*." A 29 year-old woman from the private clinic expressed that *"I wouldn't do that. I won't let them experiment with a drug on me*." A 63 year-old female from the private clinic mentioned "*No, I won't agree. I don't want anyone to experiment on me*." When told that doctors are blinded to study treatment, a 26 year-old male from the public clinic said: "*No, I'm not comfortable with it. Because we're humans and not lab animals. Because there's a degree of degradation to a human being by experimenting on them*." When asked about enrolling children in a drug clinical trial, a 40 year-old woman from the private clinic mentioned: "*No of course not. If they just want to monitor him that's okay, but they can't experiment on him*."

## Discussion

This study reveals several important initial insights regarding the perspectives of Egyptians toward medical research that might be applicable to individuals in other developing countries. First, the individuals in our sample had a good general understanding of the concept of research. They also understood that the goal of medical research is to advance medical progress that will help future patients. Also, most of the respondents would participate in medical research, even if there were no prospect of direct benefits for their participation.

This interest in participating in medical research, however, was conditional on their perceived risks associated with the research. As the interviews progressed and when respondents were asked to comment on different types of medical studies, it became apparent that many were hesitant with participating in studies that presented greater than minimal risks. For example, most participants found studies involving surveys and the giving of blood samples to be acceptable. However, the willingness to participate decreased with greater degrees of perceived risks associated with the research (e.g., endoscopy and drug trials).

In addition to concerns with risks, discomfort with drug trials might also be related to a general unfamiliarity with randomized drug trials. Furthermore, throughout the interview, when the subject of drug trials came up, many respondents would associate such studies with experimentation, to which they found to be unacceptable and degrading to humans. Finally, another factor contributing to hesitation to participate in clinical trials might be due to the realization that such trials are funded by foreign pharmaceutical companies, which might engender the notion that wealthy foreign establishments want to use Egyptians as 'guinea pigs' to test drugs that they could not otherwise test on their own citizens. Distrust of the motives of scientists from developed countries was observed in an informed consent study involving a West African country [19]. Future studies involving individuals from the Middle East should be performed to determine the presence, if any, of distrust towards foreign investigators.

We also found that several research methods caused anxiety, discomfort, and confusion among the participants. For example, many did not feel comfortable with nor understood the need for randomization. Investigators in other countries have also uncovered discomfort and misunderstanding with the concept of randomization. A study involving Danish patients with cancer and inflammatory bowel disease also found that many had a negative or a hesitate view of randomization and that several would have wanted to choose the treatment option themselves [11]. A focus-interview study of Japanese individuals also discovered that many had a feeling of repulsion towards randomization [13]. An Australian study found that many patients disliked being part of an experiment and patients' willingness to join a clinical trial was negatively associated with uncertainty of treatment allocation [20]. A study involving oncology patients found that only one-third of the respondents would consider taking part in a trial comparing different treatments where treatment was selected at random by a computer [12]. A recent qualitative interview study involving participants involved in six different randomized controlled trials involving interventions to prevent or ameliorate an infectious disease in Africa and the Caribbean found that there it was typical for participants not to know how they would be assigned to the intervention groups [14].

Many participants in our study sample were also confused and concerned with the concept of doctors being blinded to their study drug assignment. Only two participants realized the scientific value of blinding in a research study. The most difficult concept for the participants to understand was that of clinical equipoise. Several participants expressed their preferences for either taking the experimental or the approved drug. More importantly, most participants expressed disbelief that their doctors would not know which study drug would be better. In a previous study involving oncology patients, investigators found that approximately one in four patients thought that the doctor would really know which study drug was better in a clinical trial and nearly three quarters thought that the doctor would ensure that they would get the study drug that was more efficacious [12]. Cassileth and colleagues also found that a high proportion of patients believed that doctors really know which of the study drugs was best [21]. Other studies have also observed that patients think that the experimental drug might be better and hence, unwilling to be randomized to a placebo arm of a clinical trial [13,22]. Such confusion regarding equipoise might also explain why there was difficulty understanding the requirement for randomization and the need for research to be designed to determine the relative efficacy of drugs.

Several participants in our study associated scientific design methods (i.e., randomization and blinding) with "experimentation," a word that imparted a negative view of research. The use of such research methods was seen as being a barrier to their participation in research. It is important that investigators are aware of these attitudes and try to enhance patient understanding of the purposes of research methods. Fallowfield and colleagues found that further information can increase willingness to participate in clinical trials [23]. However, other studies have documented that research subjects do not understand research concepts even when research staff specifically explain these concepts and practices to them [9,19,24]. The failure to understand these important research design methods has strong implications on obtaining a valid informed consent.

Difficulty understanding and accepting the concept of blinding and equipoise might be due to the phenomenon of the therapeutic misconception [25,26], whereby participants in a research study believe that they are receiving best medical care. Such beliefs would make it difficult to believe that there are no differences between the study interventions and that their doctor would not know which study drug would be best for them. Indeed, we uncovered subtle indicators of the therapeutic misconception throughout the interviews. For example, participants thought that medical research represents treatment or has the same goal as medical care. Specifically, several participants expressed a greater willingness to volunteer for research if there were benefits to themselves or their children. Other studies have also found that patients enrolled in drug trials expect to receive personal benefits, despite receiving contrary information [14,27]. Studies involving patients from Western countries also reveal their discomfort with randomization, blinding, and equipoise, which has been attributed to the therapeutic misconception and with patients believing that physicians would know "what is best" for their patients [8,11,12].

Our results regarding the informed consent process also question the existence of any sharp division between developed and developing countries. A principle of Western ethics is that the individual acts as an autonomous agent, whereas in many cultures in developing countries, the individual is viewed in the context of the social group [28-30]. Accordingly, it has been questioned whether it is possible to have one internationally agreed standard of informed consent in medical research, because the concept of individuals making personal decisions independent of the family or the community is not common to all cultures [28,30]. However, all of the participants in our study valued the concept of informed consent, as they thought their permission to be in a research study was paramount. Several participants expressed this concept in the language of rights. Studies on informed consent from other developing countries have also shown that individuals participating in research desire to make their own decisions regarding participation [19,31]. We also found little discomfort with the concept of providing a written signature on informed consent forms, as only two participants found signing such documents threatening. This observation is contrary to the general viewpoint that insistence on having a signed consent form might not always be appropriate for many individuals in developing countries [2,27,32].

However, rather than being dependent on a concept of a homogeneous culture existing in any one region or country, notions of autonomy or the acceptability of written informed consent may very well depend on other factors within a region, such as varying education levels and the different degrees of modernity that might exist between urban and rural communities, which might reflect the uneven process of globalization of ideas and practices that has otherwise facilitated convergence between different cultures [33].

For example, until recently, Egyptians were only asked to give their signatures for birth certificates, marriage certificates, identification documents, and during buying and selling property. Accordingly, the requirement for a signature on other written documents had been viewed with suspicion, particularly if the signatory is illiterate and cannot validate the contents of the document. Now however, particularly in urban areas and among those with a higher level of education, more people are opening bank accounts, getting loans and using credit cards, all of which require the use of signatures (personal communication: authors SE-K and SSK). Hence, our results might have been due to the inclusion of participants with higher educational levels and who have been exposed to financial practices that are more modern compared with those in rural areas of Egypt.

Hence, instead of culture itself, which is increasingly being viewed as internally heterogeneous and dynamic [33], perspectives on medical research might be more reflective of the specific local context within any one country. This notion of the heterogeneity of cultures has important implications for institutional review boards that review the acceptability of the conduct of research in heterogeneous communities.

Our results showed a mixed reaction regarding the acceptance of proxy consent for research participation when in a critically ill state and one could not decide for themselves. Egyptian society is a family-oriented society and hence, one might expect that family decision making would be acceptable, similar to what occurs in other developing countries [31,34]. Our seemingly contrary results might be explained by proxy consent being a novel concept in a society in which individuals are not often asked to participate in drug trials. The finding that many women would refuse enrollment via a proxy consent mechanism might be attributed to the high education level of our study sample, which might lead to a more equal power relationship between health professionals and patients. Results might differ in rural areas of Egypt where the influence of family elders or male authority figures are more dominant in health care decision making [31,35]. A majority of the participants were not comfortable with enrolling children in medical research. Many cited a concern with the risks or stressed there needed to be some assurance of benefits before having their child enrolled in research. This concern with risks and benefits regarding children participation was shown in another study involving participants in a developing country [27].

An important theme that emerged in many aspects of the interviews was the importance of trust. Indeed, many participants expressed that their participation in medical research requires a trusting relationship between patients and physicians and several expressed that such trust already exists with their physicians. Such findings make clear that a relationship built on trust with the medical care provider is essential not only to medical care, but also to future medical research endeavors in Egypt. The existing trust that Egyptians have with their physicians was validated when most of the participants did not think that physicians performed research only to advance their careers. Many who thought that career advancement was a motivating for the conduct of research also thought that this was acceptable, because they believed that investigators are also motivated to advance medicine and help future patients. Accordingly, clinical investigators in Egypt should anticipate a large degree of trust and hence, willingness to participate in medical research on the part of most patients. One concern associated with a large degree of trust is that patients might adopt a more passive role in the informed consent process and seek less information regarding the study.

There are several limitations to this study. First, our sample was limited to those who agreed to participate in a survey study and hence, it is possible that those who rejected participation would add different insights regarding the acceptability of research and the existence of trust occurring in Egyptian society. Second, our sample size was small and hence, our results might not be generalizable to other parts of Egypt as well as to other Middle Eastern countries. Indeed, our sample was more representative of the culture and attitudes of the northern Nile Delta region; varying attitudes might exist among the Sa'ayda and Noubian Egyptians in southern (Upper) Egypt and the Bedouins in Sinai. Third, the education level of our participants was higher than the median level of Egyptians from other parts of the country. As discussed above, differences in culture, education, and socioeconomic background among different areas in Egypt might have influenced the results regarding written informed consent, views about individualized decision making, and the understanding of the general concept of research. Despite the high education levels of our sample, many still had difficulty understanding basic research design concepts. Such difficulty would be even more extensive in rural areas, where higher illiteracy rates exist. Also, despite the small sample size, certain perspectives revealed regarding medical research, e.g., the reluctance to assume greater than minimal risk, the concerns with drug clinical trials, and the importance of trust in decisions regarding participation, might reflect the general attitudes of Egyptian society and those in other developing countries [13,14,27]. As qualitative research methodology is used primarily to generate, rather than test hypotheses, our results will serve as the basis for future studies incorporating highly structured surveys for broader distribution.

## Conclusion

While our findings require validation in a larger sample that includes other areas in Egypt and other developing countries, we are able to make several recommendations. First, there needs to be enhanced educational efforts directed towards the lay public on fundamental issues regarding research design concepts, as well as the distinction between research and medical care. Researchers should also receive additional training on explaining research concepts in understandable language. Further empirical research is needed to measure the effect of creative interventions to improve participants understanding of these concepts. These issues have strong implications for achieving a valid informed consent.

Second, individuals are fearful of the risks inherent in medical research and therefore, investigators and members of research ethics committees need to give assurances that procedures will be in place that minimize these risks and enhance the protection of their rights and welfare. Third, many patients and members of the lay public recognize the value of informed consent and the concept of them providing their permission for their participation in medical research. Accordingly, undergraduate and graduate curricula, and especially medical and paramedical school training, should include familiarization with the concepts of research ethics and the need for the valid, informed consent of participants before their enrollment into any research project.

Finally, investigators need to know that individuals are willing to participate in medical research, because they recognize the value of such research towards advancing the field of medicine and they are trustful of the medical profession. However, such trust might be fragile and hence, a strong system of research ethics education and ethics review would help ensure that such trust is maintained.

## Competing interests

The author(s) declare that they have no competing interests.

## Authors' contributions

SSK made substantial intellectual contributions to this study. She was involved in the conception and design of the overall study and the survey tool, she conducted the interviews and helped transcribed the interviews, helped with the analysis of the results and was significantly involved in the drafting and writing of the paper. MR made substantial intellectual contributions to this study. She was involved in the conception and design of the overall study and the survey tool, she conducted the interview and helped transcribe the interviews and have been involved in reviewing the manuscript. HJS served as senior author on this manuscript and made substantial intellectual contributions to this study. He was involved with the conception and design of the overall study and the survey tool; he gave advice on the conduct of the interviews, and he made significant contributions to the analysis of the results and made substantial intellectual contributions to the writing of the manuscript. SE-K made significant comments regarding Egyptian culture and made contributions to the writing of the final draft of the manuscript. ME-S was involved in the initial discussions of the research project and reviewed several drafts of the manuscript. All authors read and approved the final manuscript.

## Pre-publication history

The pre-publication history for this paper can be accessed here:


